# A homolog of cyclophilin D is expressed in *Trypanosoma cruzi* and is involved in the oxidative stress–damage response

**DOI:** 10.1038/cddiscovery.2016.92

**Published:** 2017-02-06

**Authors:** Patricia L Bustos, Bibiana J Volta, Alina E Perrone, Natalia Milduberger, Jacqueline Bua

**Affiliations:** 1 Instituto Nacional de Parasitología ‘Dr. Mario Fatala Chabén’- A.N.L.I.S. Malbrán, Av. Paseo Colón 568, C1063AC S, Buenos Aires, Argentina; 2 Consejo Nacional de Investigaciones Científicas y Técnicas, Argentina; 3 CAECIHS, Universidad Abierta Interamericana, Av. Montes de Oca 745, 2º piso, C1270AAH, Buenos Aires, Argentina

## Abstract

Mitochondria have an important role in energy production, homeostasis and cell death. The opening of the mitochondrial permeability transition pore (mPTP) is considered one of the key events in apoptosis and necrosis, modulated by cyclophilin D (CyPD), a crucial component of this protein complex. In *Trypanosoma cruzi*, the protozoan parasite that causes Chagas disease, we have previously described that mitochondrial permeability transition occurs after oxidative stress induction in a cyclosporin A-dependent manner, a well-known cyclophilin inhibitor. In the present work, a mitochondrial parasite cyclophilin, named *Tc*CyP22, which is homolog to the mammalian CyPD was identified. *Tc*CyP22-overexpressing parasites showed an enhanced loss of mitochondrial membrane potential and loss of cell viability when exposed to a hydrogen peroxide stimulus compared with control parasites. Our results describe for the first time in a protozoan parasite that a mitochondrial cyclophilin is a component of the permeability transition pore and is involved in regulated cell death induced by oxidative stress.

## Introduction

Mitochondria serve as the ‘powerhouse’ that provides near 90% of ATP necessary for cell life. However, studies over the past 30 years have provided strong evidence that mitochondria also have a central role in cell death in response to oxidative stress.^1^ Accumulation of reactive oxygen species (ROS) together with Ca^2+^ overload induces the mitochondrial permeability transition (mPT), which indicates an abrupt increase in the permeability of the inner mitochondrial membrane to small solutes.^2,3^ In this setting, osmotic forces drive a massive entry of water into the mitochondrial matrix, leading to an immediate cessation of the bioenergetics and biosynthetic functions of mitochondria that depend on the transmembrane potential (ΔΨm) and the release of multiple mitochondrial proteins, including various activators of the intrinsic pathway of apoptosis into the cytoplasm.^4^

The occurrence of mPT is the key event of the regulated cell death process. The term ‘regulated’ refers to cases that can be inhibited by specific pharmacological or genetic interventions, implying that they rely on defined molecular machinery, though sometimes known to partial extents. Until recently, apoptosis was considered as the only form of regulated cell death but since late 1990s it has become clear that necrosis can occur in a regulated fashion, known as regulated necrosis (RN). For example, the mPT represents one of the major gateways to mitochondrial apoptosis, a process that is mediated and regulated by proteins of the Bcl-2 family, namely, mitochondrial outer membrane permeabilization (MOMP).^5,6^ However, the mPT is also capable of triggering a peculiar process of RN that critically relies on peptidyl prolyl isomerase F (PPIF, best known as cyclophilin D, CyPD), which results in the opening of a non-selective pore, namely the mitochondrial permeability transition pore (mPTP). The importance of CyPD for mPT has been recognized for a long time mostly due to the consistent cytoprotective effects mediated *in vitro* and *in vivo* by the pharmacological CyPD inhibitor cyclosporin A (CsA).^2,3^ Nowadays, CyPD remains the only confirmed functional constituent of the mPTP.

Moreover, both the administration of CsA and the *Ppif*^*−/−*^genotype have been shown to limit necrotic cell death, *in vitro* as well as *in vivo*, in a variety of pathophysiological settings including ischemia-reperfusion injuries of the heart,^7–9^ brain^10,11^ and kidneys.^12,13^ Interestingly, the pro-necrotic activity of CyPD appears to be regulated by a mitochondrial pool of the oncosupressor protein p53.^11^ However, the precise molecular mechanisms that execute mPT-dependent RN is still obscure. It remains to be formally demonstrated whether CyPD mediates RN as part of the mPTP or independently from the assembly of such a protein complex. As mentioned above, so far no mPTP components other than CyPD have been shown to be indispensable for mPT *in vivo*. This may indicate that CyPD can assemble the mPTP in a relatively unspecific manner or that CyPD exerts mPTP-independent lethal functions.

*Trypanosoma cruzi* is a unicellular protozoan parasite which infects 7–8 million people in South America as well as in other parts of the world through migrations from endemic areas.^14^ The *T. cruzi* infection can evolve into Chagas disease, a potential life-threatening illness.^15^

We have previously described the *T. cruzi* *CyP* gene family, which consists of 15 paralogs.^16,17^ Analyses of clusters formed by the *T. cruzi* CyPs with others encoded in various genomes revealed that eight parasite cyclophilins (*Tc*CyP19, *Tc*CyP21, *Tc*CyP22, *Tc*CyP24, *Tc*CyP35, *Tc*CyP40, *Tc*CyP42 and *Tc*CyP110) have orthologs in many different genomes (reviewed in Galat and Bua^18^). Moreover, 4 *T. cruzi* CyPs (19, 22, 28 and 40 kDa proteins) were isolated by CsA affinity from parasite lysates that proved to have PPIase activity, inhibited by CsA.^16,17^

We have also described a regulated cell death process occurring under oxidative stress conditions in *T. cruzi*, including features as phosphatidyl serine exposure, DNA degradation and cytochrome *c* translocation into the cytosol, among others. All these events were sensitive to CsA inhibition, suggesting that a *T. cruzi* mitochondrial cyclophilin could be present in a mPTP–like in this protozoan.^19^

In the present work, we demonstrate the existence of mitochondrial cyclophilin*, Tc*CyP22, homolog to the mammalian CyPD, in *T. cruzi* and described its specific role in parasite cell death in response to oxidative stress.

## Results

### A mitochondrial cyclophilin is expressed in *T. cruzi*

We have previously described that some cell death features induced by oxidative stress could be inhibited by cyclosporin A in *Trypanosoma cruzi*, thus indicating that a parasite mitochondrial CyP may be involved in these events.^19^

To predict the CyPs with putative mitochondrial localization within the parasite cyclophilin family, we performed a bioinformatic analysis, combining the information obtained from analyzing the amino acid sequences of the CyP family with two different servers, iPSORT and MitoProtII v1.101 (see Material and Methods section). The parameters obtained for each CyP are listed in Supplementary Table S1. We observed that *Tc*CyP21, *Tc*CyP22, *Tc*CyP24 and *Tc*CyP25 presented high probability of being localized to *T. cruzi* mitochondrion.

When we aligned these protein sequences with human CyPD CyPD (GenBankAcc No. P30405), *Tc*CyP22 (GenBankAcc No. AY349021.1/TriTryP DB Acc No. TcCLB.510259.50) presented the highest percentage of amino acid identity (65%) and similarity (77%) (Supplementary Figure S1).

**Figure 1:**
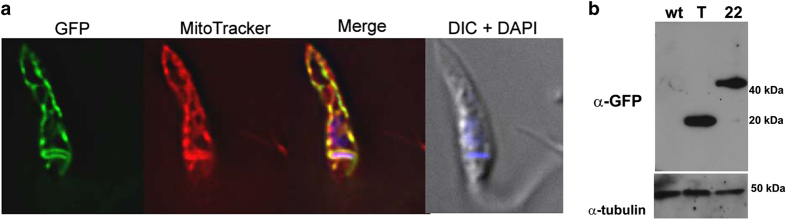
Expression and subcellular localization of *Tc*CyP22 in transgenic epimastigotes. (**a**) Monoclonal antibodies against GFP were used to detect *Tc*CyP22 (green). Co-localization with MitoTracker (red) was observed. Merge images show the co-localization in yellow. DAPI was used to stain nucleus and kinetoplast (blue). DIC, differential interference contrast microscopy. (**b**) Antibodies against GFP detected unique bands of the expected sizes in parasite lysates. Antibodies against α1-tubulin were used to confirm equal loading. 22, epimastigotes carrying the pTREX-*Tc*CyP22-GFP; T, epimastigotes carrying the pTREX-GFP empty vector, as a control; wt, wild-type epimastigotes.

Since some cross-reactivity might appear with antibodies against conserved regions of cyclophilins in indirect immunofluorescence techniques, we developed a molecular biology strategy to confirm the *Tc*CyP22 localization. The complete DNA sequence was amplified by PCR and cloned with a C-terminal enhanced version of the green fluorescent protein (eGFP) in the pTREX vector. A transgenic parasite line carrying this construction was generated, hereinafter referred to as *Tc*CyP22-OE. Immunofluorescence was performed with monoclonal antibodies against GFP in *Tc*CyP22-OE in the epimastigote stage. *Tc*CyP22 localized to the mitochondrion, as demonstrated by co-localization with MitoTracker staining (Figure 1a). To investigate if *Tc*CyP22 was expressed in the other stages of the parasite lifecycle, we infected VERO cells. We obtained transgenic parasites in the amastigote and tripomastigote stages. We observed that *Tc*CyP22 was expressed and localized to the mitochondrion of both stages as well (data not shown).

Protein size was confirmed by Western blot analysis of total parasite lysates, where a unique band was detected in each lane, using antibodies against GFP (∼40 kDa for *Tc*CyP22-GFP and ∼20 kDa for GFP; Figure 1b).

### *Tc*CyP22-overexpressing parasites are more susceptible to oxidative stress damage

As we intended to use the transgenic parasites in the epimastigote stage for further studies, we first attempted to evaluate if the overexpression of *Tc*CyP22 altered parasite viability. We confirmed that there were no significant differences in the number, motility and morphology between *Tc*CyP22-OE and wild type parasites (data not shown).

Since the specific mitochondrial cyclophilin modulating these events remained unknown, we challenged *Tc*CyP22-OE parasites in oxidative stress conditions to analyze if *Tc*CyP22 is involved in cell death.

### *Tc*CyP22-OE shows enhanced loss of mitochondrial membrane potential (ΔΨm)

The loss of mitochondrial membrane potential (ΔΨm) occurs at the onset of cell death process. To study the ΔΨm of *Tc*CyP22-OE in oxidative stress conditions, parasites were loaded with the mitochondrial fluorescentΔΨm indicator MitoTracker. Parasites carrying the empty pTREX-GFP vector were used as control. After oxidative stress induction, transgenic epimastigotes were analyzed by flow cytometry to evaluate the fluorescence intensity, which was considered proportional to the ΔΨm of the parasite population at different time points.

**Figure 2:**
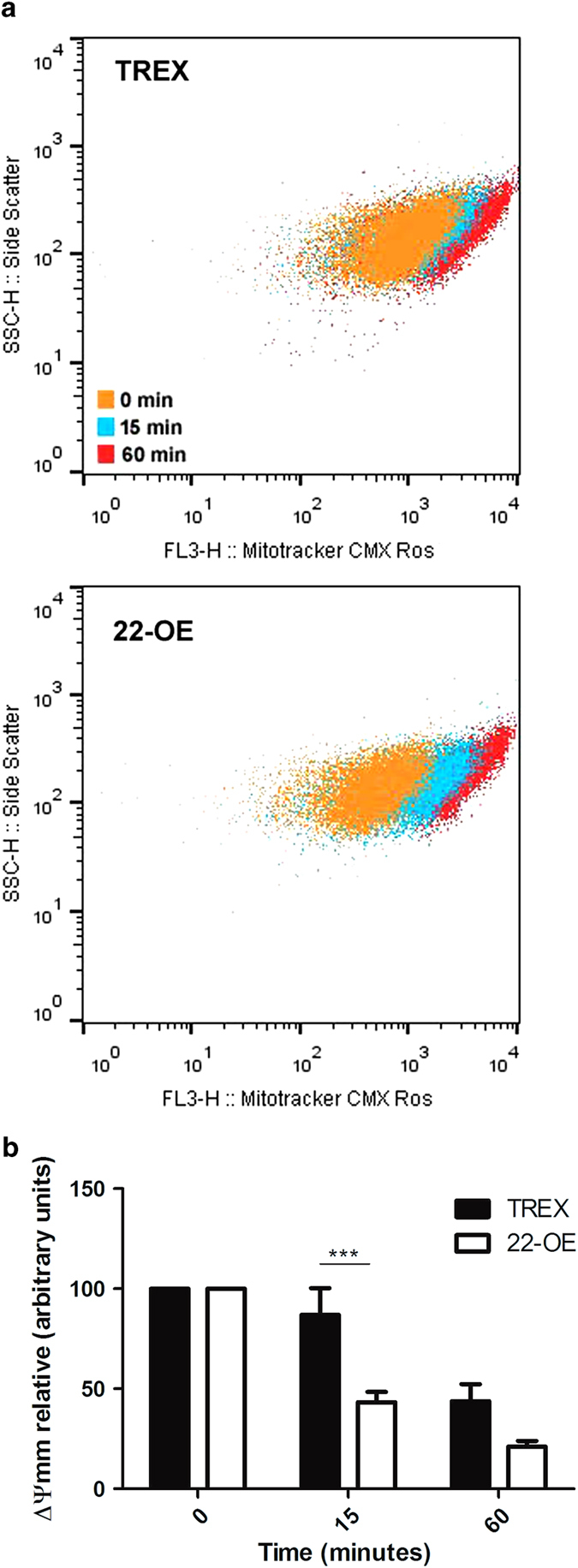
Loss of mitochondrial membrane potential (ΔΨmm) in oxidative stress conditions. Parasites were loaded with the ΔΨmm fluorescent indicator, MitoTracker. Oxidative stress conditions were generated with 5 mM H_2_O_2_. At 15 and 60 min, parasites were analyzed by flow cytometry. In (**a**), dot plots of a representative experiment are shown. (**b**) Quantification of three independent experiments. TREX, Control parasites carrying the empty pTREX-eGFP vector; 22-OE, transgenic parasites overexpressing *Tc*CyP22-GFP (means±s.d., *n*=3, ***P<0.001 (22-OE *versus* TREX at 15 min), two-way – RM – ANOVA).

The fluorescence measured at the beginning of the induction with H_2_O_2_ (time 0 min) was considered as 100% ΔΨm (in arbitrary units). After 15 min, a substantial decrease to 43.26% was observed in *Tc*CyP22-OE parasites. In contrast, ΔΨm in control parasites (TREX), at 15 min lowered only to 86.9%. When measured at 60 min, loss of ΔΨm was enhanced in *Tc*CyP22-OE compared with TREX parasites, decreasing to 21.0 and 43.26%, respectively (Figure 2). *Tc*CyP22-OE parasites evidenced an enhanced loss of ΔΨm compared with control parasites in oxidative stress conditions.

### The genomic DNA of *Tc*CyP22-OE parasites results highly damaged after oxidative stress induction

To further characterize the damage caused by oxidative stress in *Tc*CyP22-OE parasites compared with control parasites, we evaluated the integrity of genomic DNA after the oxidative stress with hydrogen peroxide by terminal deoxynucleotidyltransferase dUTP nick end labeling (TUNEL).

**Figure 3:**
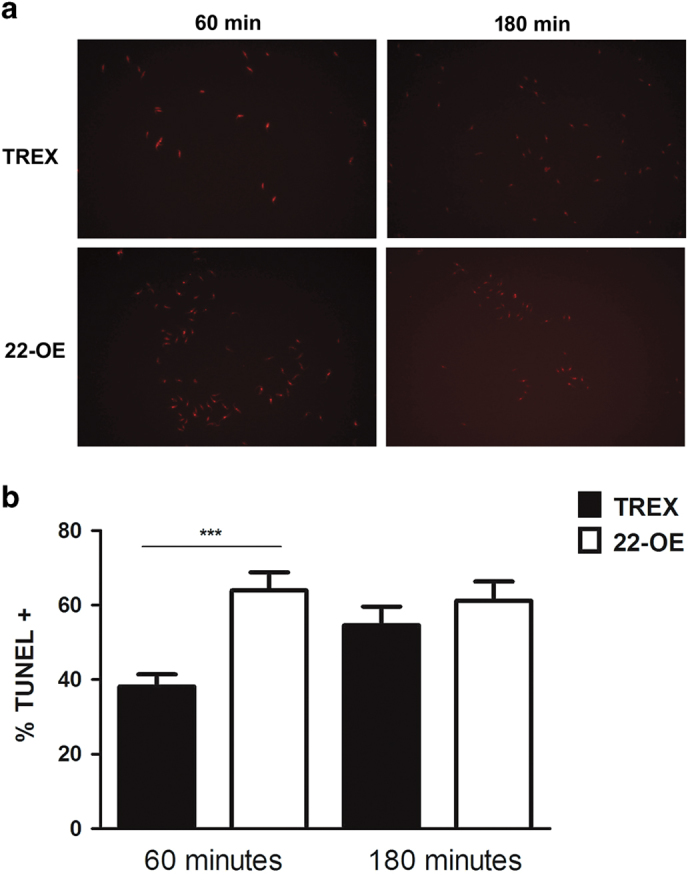
DNA damage in oxidative stress conditions. Oxidative stress conditions were generated with 5 mM H_2_O_2_. At 60 and 180 min, parasites were fixed and stained for TUNEL, according to manufacturers’ specifications. (**a**) Fluorescence images of a representative experiment. TUNEL^+^ parasites present nuclei with red staining. (**b**) Quantification of TUNEL^+^ parasites, expressed as percentage over total parasites for each condition. TREX, Control parasites carrying the empty pTREX-eGFP vector; 22-OE, transgenic parasites overexpressing *Tc*CyP22-GFP (means±s.d., *n*=3, ***P<0.001 (22-OE *versus* TREX at 60 min), Student’s *t*-test).

We evaluated the percentage of positive TUNEL-stained parasites at 60 and 180 min after oxidative stress. At 60 min, we observed that *Tc*CyP22-OE presented a significantly higher percentage of TUNEL^+^ than TREX (63,93% *versus* 38,18%, respectively). However, the percentage of TUNEL^+^-*Tc*CyP22-OE remained higher than TUNEL^+^-TREX control parasites, the difference was not significant after 180 min (Figure 3).

### An increased number of *Tc*CyP22-OE parasites presents loss of plasma membrane integrity

To evaluate another cell death feature, we used propidium iodide (PI), which positively stains the parasites that have lost their plasma membrane integrity. At different time points, parasites were washed to remove H_2_O_2_, stained with PI and analyzed by flow cytometry. The fluorescence intensity was used to identify PI^+^ parasites.

**Figure 4:**
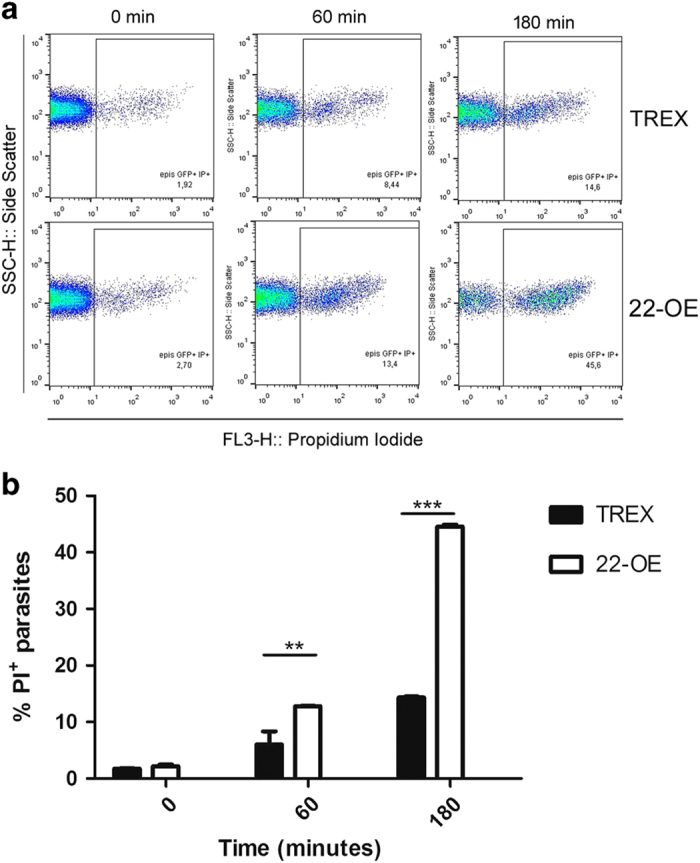
Loss of plasmatic membrane integrity during oxidative stress conditions. Parasites were incubated with 5 mM H_2_O_2_. At 60 and 180 min, parasites were stained with propidium iodide and analyzed by flow cytometry. In (**a**), dot plots of a representative experiment are shown. (**b**) Quantification of three independent experiments. TREX, Control parasites carrying the empty pTREX-eGFP vector; 22-OE, transgenic parasites overexpressing *Tc*CyP22-GFP (means±s.d., *n*=3, **P<0.01 (22-OE *versus* TREX at 60 min); ***P<0.001 (22-OE *versus* TREX at 180 min), Two-way – RM – ANOVA).

Since loss of membrane integrity is an event that seems to occur at the end of cell death process, after 60 min under oxidative stress stimulus only a low percentage of PI^+^ parasites were observed, although significantly higher in *Tc*CyP22-OE than in TREX control parasites (12 and 6%, respectively). After 180 min, the proportion of PI^+^-*Tc*CyP22-OE parasites increased, reaching 44.55% of the population, compared with 14.35% in TREX control parasites (Figure 4), indicating that *Tc*CyP22-OE parasites present a higher sensitivity to the oxidative stress environment.

### Phospatidylserine exposure is not a significant cell death feature neither in *Tc*CyP22-OE or TREX control parasites

With the aim of distinguishing the cell death process that occurs in response to H_2_O_2_ in *Tc*CyP22-OE parasites, we measured phosphatidylserine (PS) exposure on the external leaflet of the plasma membrane. This event is evidenced by the annexin V binding assay to detect apoptotic cell death. Herein, we used a double staining procedure, which consists in using annexin V conjugated to phycoerythrin (AV-PE) to detect PS exposure and the fluorescent 7-aminoactinomycin D (7-AAD), for plasma membrane integrity, that allows the identification of different sub-populations: AV-PE^+^ 7AAD, considered as early apoptotic parasites; AV-PE^-^ 7AAD^+^ as necrotic ones. However, double positive AV-PE^+^ 7AAD^+^ parasites cannot be unequivocally distinguished as late apoptotic or necrotic.

**Figure 5:**
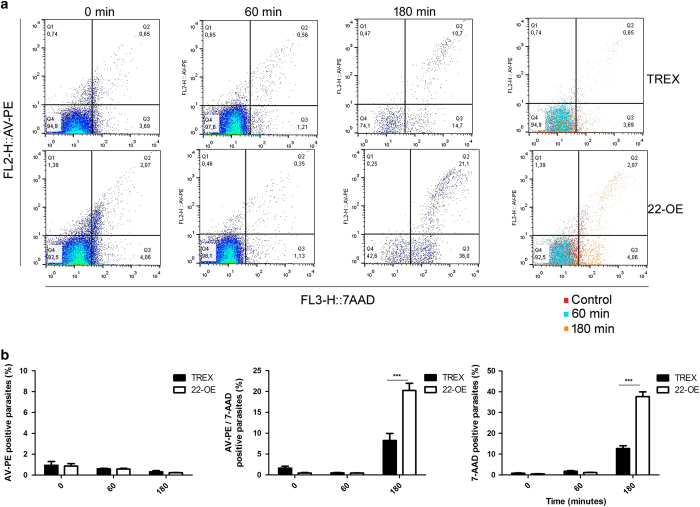
Phosphatidylserine exposure during oxidative stress. Parasites were incubated with 5 mM H_2_O_2_. At 60 and 180 minutes, parasites were stained with annexin V-phicoeritrin and 7AAD and analyzed by flow cytometry. Heat-shock treated parasites were used as positive control. In (**a**), dot plots of a representative experiment are shown. (**b**) Quantification of three independent experiments. TREX, Control parasites carrying the empty pTREX-eGFP vector; 22-OE, transgenic parasites overexpressing *Tc*CyP22-GFP ***P<0.001 (22-OE *versus* TREX at 180 min), two-way – RM – ANOVA).

When oxidative stress was generated with 5 mM H_2_O_2_, no significant AV-PE^+^ staining was observed for *Tc*CyP22-OE or TREX parasites, indicating that an apoptotic-like cell death would not be occurring in these parasites, under the conditions assayed. However, we were able to detect 7AAD^+^ parasites (either simple or double positives), which indicates the plasma membrane integrity was lost. According to the results obtained with PI staining in parasites incubated with H_2_O_2_ for 180 min (Figure 4), *Tc*CyP22-OE showed a significantly higher percentage of 7-AAD^+^ sub-population (37.7+20.23%) than TREX control parasites (12.7+8.23%; Figure 5).

The results described in this work suggest that *Tc*CyP22-OE parasites undergo a regulated necrosis rather than an apoptotic-like cell death, in response to hydrogen peroxide. Under the conditions described, the parasites overexpressing the mitochondrial cyclophilin *Tc*CyP22 presented an enhanced sensitivity to oxidative stress environment.

## Discussion

Cyclophilin D (CyPD) is a peptidyl prolyl isomerase (PPIase) localized to the mitochondrial matrix of mammalian cells. CyPD has been widely studied as the central component of the mitochondrial permeability transition pore (mPTP) in mammalian cells, a key channel that is formed during stress conditions such as Ca^2+^ overload and increased ROS production and that is capable of triggering a peculiar process of regulated necrosis which critically relies on CyPD.^4^ Although the specific molecular structure of the mPTP remains under discussion, CyPD has been the unique component proven to be indispensable, either *in vivo*, in animals with CyPD genetic ablation (Ppif^−/−^) and *in vitro*, with CyPD pharmacological inhibition with cyclosporin A.

As it was mentioned above, our research group has described the *T. cruzi* CyP gene family and reported the expression of several parasite cyclophilins that exhibited enzymatic PPIase activity, inhibited by CsA.^16,17^ Recently, we observed that parasites submitted to an oxidative stress environment with 5 mM H_2_O_2_, underwent cell death events such as DNA degradation, ROS production, cytochrome *c* translocation, which occurred after induction and were sensitive to CsA inhibition, suggesting that a *T. cruzi* mitochondrial cyclophilin could be present in a mPTP–like in this protozoan.^19^

In the present report, we identified that a homolog of mammalian cyclophilin D is expressed in *T. cruzi,* named as *Tc*CyP22. This protein was localized to parasite mitochondrion in three stages of the parasite life cycle as expected, and to our knowledge, this is the first identification of a homolog of a CyPD in a protozoan parasite.

An overexpression of *Tc*CyP22 cyclophilin in the epimastigote stage (*Tc*CyP22-OE parasites) was our strategy to study the role of this protein in oxidative stress conditions, evaluating different known cell death features.

The loss of the mitochondrial membrane potential (ΔΨm) occurs at the onset of a cell death process and was measured in *Tc*CyP22-OE parasites during oxidative stress; TREX parasites, carrying the empty pTREX-GFP vector, were used as control parasite population.

The observation that the loss of ΔΨm was significantly increased in *Tc*CyP22-OE parasites with respect to TREX control parasites, when measured at 15 and 60 min after the addition of 5 mM H_2_O_2_ (Figure 2), indicates that the overexpression of *Tc*CyP22 resulted in an enhanced sensitivity of parasites to the oxidative stress stimulus. In *T. cruzi*, the loss of ΔΨm in response to oxidative stress had already been studied using the fluorescent indicators JC1^(ref. 20)^ and safranine-O.^20,21^ However, in our experimental model, these dyes could not be used, given the overlap with GFP signal.

Oxidative DNA damage is an unavoidable consequence of cellular metabolism. However, in cells undergoing oxidative stress caused by exogenous compounds, DNA damage can arise through overproduction of ROS and when lesions cannot be removed, chronic DNA damage triggers specific cell death responses such as apoptosis^22^ or some type of regulated necrosis.^4^

When *Tc*CyP22-OE parasites were exposed to 5 mM H_2_O_2,_ a significantly higher DNA damage could be observed after 60 min by TUNEL technique, compared with the DNA damage observed in TREX control parasites (Figure 3).

DNA damage has also been reported to occur in other protozoan parasites, as *Leishmania donovani* under oxidative stress caused by 3 mM H_2_O_2_^(ref. 23,24)^ and in *T. cruzi*, with 20% (V/V) fresh human serum,^20^ and with of 5 mM H_2_O_2_ in our previous study.^19^

Regarding parasite viability, during the whole oxidative stress induction, *Tc*CyP22-OE presented an enhanced sensitivity to H_2_O_2_ stimulus in comparison with TREX control parasites. A significantly higher number of *Tc*CyP22-OE parasites lost their plasma membrane integrity, evidenced by the two different fluorescent indicators assayed, propidium iodide (Figure 4) and 7-AAD (Figure 5).For example, after 180 min of oxidative stress induction, *Tc*CyP22-OE parasites PI^+^ were ∼45% (Figure 4) and 58% could be detected with 7-AAD (Figure 5). In contrast, for TREX control parasites these percentages were significantly lower, being ∼15% for PI^+^ (Figure 4) and ∼20% with 7-AAD^+^ (Figure 5). PI is normally used as the viability probe of choice in flow cytometry and strongly intercalates DNA depending on the degree of unwinding. However, the viability stain 7-AADis a fluorescence DNA-binding agent that intercalates between cytosine and guanine bases and has a high DNA-binding constant and a slow post-fixation and permeabilization dissociation rate.^23,24^

With the aim of discriminating the cell death process that might be occurring in *Tc*CyP22-OE, we intended to detect phosphatidylserine (PS) exposure, which is a hallmark of apoptotic cell death, using the annexin V binding (AV-PE), together with 7-AAD staining. However, no significant single AV-PE^+^ parasites could be detected during the whole time of the oxidative stress assay, nor for *Tc*CyP22-OE or TREX control parasites (Figure 5), thus indicating that an apoptotic-like cell death would not be the type of cell death occurring in these conditions.

In the literature, PS exposure has been reported in *T. cruzi*, with different approaches. For instance, PS exposure was positively detected after oxidative stress induction using 20% (v/v) FHS, in Tulahuen strain, grown in BHI.^20^ PS exposure was also detected in axenic cultures of Tulahuen, grown in Diamonds’ medium^25^ and in our previous work, in Brener, grown in BHI culture medium.^19^

Nevertheless, when grown in LIT medium, PS exposure was only detected in the Y strain trypomastigote stage but not in epimastigotes and amastigotes,^26^ in agreement with our results with the same *T. cruzi* strain and medium culture.

Nowadays, the study of cell death pathways has become a complex area, where every cell death process appears to be overlapped with the others, rather than independent or non-connected mechanisms. In metazoans, the signaling pathways leading to regulated necrosis exhibit a crosstalk with the molecular cascades that lead to apoptosis, either due to shared signal transducers or for the existence of negative feedback circuitries, where one cell death process (most often apoptosis) actively inhibits the other (most frequently necrosis). Regulated necrosis is not only closely interconnected with apoptosis and autophagy, but also exhibits an elevated degree of overlap among different necrotic sub-routines, which would explain why the inhibition of a single signaling pathway is not enough to provide sufficient cytoprotective effects.^4^

Regarding *T. cruzi*, it could be expected that cell death pathways carried out by this single-cell organism were less complex than in metazoans. However, diversity still can be found in this protozoan parasite.^19–21,25^ The co-existence of several, still undescribed mechanisms underlying the mPT caused by oxidative stress conditions in *T. cruzi* could explain the different outcomes for the variety of stimuli assayed.

Taken together, results shown in this work indicate that *Tc*CyP22 is not only localized to parasite mitochondrion, as it was expected for a homolog gene of mammalian CyPD, but also that parasites overexpressing this mitochondrial cyclophilin displayed an enhanced sensitivity to oxidative stress conditions compared with TREX control parasites, which leads to increased cell death, as has been reported for mammalian systems with CyPD overexpression.^7^

Our results encourage further experiments to continue elucidating the role of *Tc*CyP22 in the metabolism of this protozoan parasite and to expand the research to other kinetoplastids.

The characterization of the pathway involving cell death in trypanosomes will lead to important insights into the biology of these parasites, the evolution of cell death pathways, and ultimately novel targets for anti-parasitic intervention.

## Materials and Methods

### Bioinformatic analysis

To identify the putative mitochondrially localized cyclophilin within the *T. cruzi* family, CyP protein sequences were analyzed using two different servers, iPSORT^27,28^ and MitoProtII v1.101,^29^ which are available on the web. iPSORT is a subcellular localization site predictor for N-terminal sorting signals (http://ipsort.hgc.jp/). MitoProtII calculates the N-terminal protein region that can support a Mitochondrial Targeting Sequence and the cleavage site (https://ihg.gsf.de/ihg/mitoprot.html).

The *Tc*CyPs identified as with the highest probability to be localized to the parasite mitochondrion were aligned with human CyPD protein sequence (GenBankAcc No. P30405) using ClustalW Multiple Alignment functions of BioEdit program (http://www.mbio.ncsu.edu/bioedit/bioedit.html).

### Cloning of *Tc*CyP22-GFP

To construct C-terminally enhanced GFP-tagged *Tc*CyP22 for localization in *T. cruzi*, the full-length cDNA of *Tc*CyP22 was amplified from *T. cruzi* genomic DNA (Y strain) by PCR using the forward primer 5′- CCCTCTAGAATGTTTTCTCGTACATGGTTTTGGG-3′ and the reverse primer 5′- CGAAGCTTGTTGTTTTTGACTTCACCACAGTCC-3′ (where the underlined nucleotides indicate the introduced *Xba*I and *Hin*dIII restriction sites, respectively). The PCR product was digested with *Xba*I and *Hin*dIII and then cloned in frame into the insertion sites in the pTREX-eGFP vector.^30^ The double-stranded sequences of GFP-tagged *Tc*CyP22 constructs were confirmed as correct by DNA sequencing. The pTREX-*Tc*CyP22-GFP DNA was transfected into *T. cruzi* epimastigotes, Y strain by electroporation in Cytomix buffer at 1.5 kV with 3 pulses 25 μF, ∞ resistance each and selected with 250 μg/ml G418 antibiotic, for 2 weeks approximately. Enrichment of parasites expressing *Tc*CYP22-GFP or GFP (from parasites carrying the empty pTREX-GFP, used as control parasites) was performed by GFP^+^ sorting using a Bio-Rad S3 cell sorter, Hercules, CA, USA.

### Parasite culture

*Trypanosoma cruzi* epimastigotes, Y strain, were grown at 28 °C in LIT medium^31^ supplemented with 10% fetal bovine serum (FBS) and 250 μg/ml G418 for pTREX selection. For oxidative stress experiments, epimastigotes were collected in log phase, at 3 days of growth. For *T. cruzi* trypomastigote and amastigote production, a monolayer of VERO cells was co-cultivated with a late log phase epimastigote suspension (>6 days), where some parasites in trypomastigote stage were spontaneously detected, using RPMI supplemented with 10% FBS. After ∼15 days, *T. cruzi* trypomastigotes and amastigotes were obtained from the supernatant. *Tc*CyP22-GFP expression was confirmed by immunofluorescence.

### Immunofluorescence microscopy

To determine the localization and expression of *Tc*CyP22 in *T. cruzi*, live parasites (epimastigote, trypomastigote or amastigote stage) were labeled for 30 min with Mitotracker Red CMXRos (Invitrogen, Waltham, MA, USA) at 50 nM in LIT culture medium. Parasites were washed in PBS and fixed with 4% paraformaldehyde in PBS at room temperature for 1 h. The fixed parasites were washed twice with PBS, allowed to adhere to poly-l-lysine-coated coverslips and permeabilized with 0.3% Triton X-100/PBS for 3 min. After blocking with PBS containing 3% BSA, 1% fish gelatin, 50 mM NH_4_Cl and 5% goat serum for 1 h, parasites were stained in 3% BSA/PBS with the rabbit antibody against GFP (1:1000, Thermo Fisher Scientific, Waltham, MA, USA), for 1 h. After thoroughly washing with PBS containing 3% BSA, parasites were incubated with Alexa 488-conjugated goat anti-rabbit antibody at 1:1000 for 1 h. The parasites were counterstained with 40,6-diamidino-2-phenylindole before mounting with GoldProLong Gold antifade reagent (Molecular Probes, Waltham, MA, USA). Differential interference contrast and fluorescent optical images were captured using an Olympus IX-71inverted fluorescence microscope with a Photometrix Cool Snap HQ charge-coupled device camera driven by DeltaVision software (Applied Precision, Fairfield, CT, USA).

### Western blot detection of *Tc*Cyp22-GFP

*T. cruzi* lysatesfrom *Tc*CyP22-OE, TREX or wild type parasites were separated by SDS-PAGE, carried out as described.^32^ Proteins were electrotransferred from 13.5% polyacrylamide gels to nitrocellulose membranes, which were blocked with a 5% (W/V) non-fat milk suspension for 1 h at room temperature. After incubation for 1 h with a rabbit monoclonal antibody against GFP (1:1000, Roche, Mannheim, Germany) and a 1:3000 dilution of the goat anti-rabbit immunoglobulin G conjugated with horseradish peroxidase (Jackson Laboratories, West Grove, PA, USA), proteins were visualized by chemiluminescence with an ECL Western Blotting Detection kit. For equal loading control, membranes were stripped and re-blotted with a rabbit polyclonal antibody against α1-tubulin (1:3000; Santa Cruz Biotechnology, Dallas, TX, USA).

### Induction of oxidative stress

Epimastigotes in log phase of growth were collected and washed twice with PBS-3% glucose to remove culture medium. Parasites were resuspended to 1×10^6^ epimastigotes/ml in PBS-3% glucose and oxidative stress was triggered by adding5 mM H_2_O_2_at room temperature. Then, parasites were centrifuged at 2300 rpm for10 min and washed once in PBS-3% glucose. Pellets were resuspended in PBS-3% glucose for subsequent studies.

### Mitochondrial membrane potential (ΔΨm)

Epimastigotes in a cell density of ∼1×10^6^ parasites/ml were loaded with the fluorescent ΔΨm indicator Mitotracker Red CMXRos (100 nM, Molecular Probes, Invitrogen) in LIT medium, for 30 min at 28 °C with gentle agitation. Then, parasites were washed twice with PBS-3% glucose and resuspended again in 1×10^6^ parasites per ml to proceed to oxidative stress incubation. Fluorescence was detected in a FACS Calibur equipment (Becton Dickinson and Co., Franklin Lakes, NJ, USA), were GFP fluorescence was detected in FL1-1 photodetector; and MitoTracker in FL-3. Data were analyzed using FlowJo10 software (Ashland, OR, USA). Results are expressed as the mean of fluorescence intensity of parasite population for each condition (50.000 GFP^+^ events were considered as 100%).

### *In situ* labeling of DNA fragments (TUNEL)

To evaluate DNA integrity after oxidative stress treatment, 1×10^6^ epimastigoteswere collected, washed twice and resuspended inPBS-3% glucose. After oxidative stress induction (as in 2.6), *in situ* detection of DNA fragments by TUNEL was performed using the *In Situ* Cell Death detection kit (Roche). Epimastigotes were collected, fixed in 4% formaldehyde and coated onto poly-l-lysine covered slides. Permeabilization was done with 0,1% (v/v) Triton X-100/PBS for 5 min at room temperature, followed by incubation with TdT buffer containing nucleotide mix (50 mM tetramethylrhodamine- 12-dUTP, 100 mMd ATP, 10 mM Tris-HCl, 1 mM EDTA, pH 7.6) for 1 h at 37 °C. The samples were visualized under a fluorescence microscope. The percentage of parasites showing a clearly visible nuclear staining was determined in TcCyP22-OE and TREX control parasites in different conditions by counting 100 cells in triplicate (blind-coded samples).

### FACS Analysis for Cell viability and Detection of Phosphatidylserine (PS) exposure

After oxidative stress incubation, 1×10^6^ epimastigotes were collected at different time points (0, 60 or 180 min) as in Induction of oxidative stress.

### Cell viability

parasites were stained with 0,5 μg/ml propidium iodide (PI). GFP fluorescence was detected in FL1-1 photodetector; PI, in FL-3. Results were expressed as the percentage of PI^+^ positive parasites with respect to the total parasite count in each condition.

### PS exposure

PS on parasites external surface of the plasma membrane was detected using the Annexin V: PE Apoptosis Detection Kit (Becton Dickinson and Co.) according to the manufacturer’s protocol. Co-staining of the parasites with 7-aminoactinomycin D (7-AAD) was performed, to evaluate the integrity of plasma membrane. Heat-shock treated parasites (3 min at 80 °C) were used as positive control for AV-PE and 7-AAD staining. GFP fluorescence was detected in FL1-1 photodetector; PE, in FL-2 and 7-AAD in FL-3.Results were expressed as the percentage of positive parasites in each quadrant with respect to the total parasite count in each condition.

Fluorescence was detected in a FACS Calibur equipment (Becton Dickinson and Co. TreeStar Inc, Ashland, OR, USA). Data analyzed using FlowJo10 software with 50 000 GFP^+^ events were considered as 100%.
