# Supplementary material for: A homolog of cyclophilin D is expressed in Trypanosoma cruzi and is involved in the oxidative stress–damage response
Source: Cell Death Discov. 2017 Feb 6;3:16092–. doi: 10.1038/cddiscovery.2016.92 (PMC5292771; doi:10.1038/cddiscovery.2016.92)
Supplement: Supplementary Figure S1 [file cddiscovery201692-s1.doc]

**Supplemental Figure S1**

10 20 30 40 50 60 70 80 90 100

**.**...|....|....|....|....|....|....|....|....|....|....|....|....|....|....|....|....|....|....|....|

**CyPD_human** **MLALRCGSRWLGLLSVPRSVPLRLPAARACSKGSGDPSSSSSSGNPLVYLDVDANG----------KPLGRVVLELKADVVPKTAENFRALCTGEK----**

**TcCyP21**  **-------MRYLLLSKVTQLLLLLLLMLSAAVSARADPVVTDK-----VYFDITIGD----------EPVGRVVIGLFGNDVPKTVENFKQLASGEN----**

**TcCyP22**  **--------------MFSRTWFWAQRKLPFYPINPKNP---------LVFFEISIGA----------QPAGRVEMELFKDAVPKTAENFRALCTGEKGVGR**

**TcCyP24**  **-----MPPRFFSAFLFGGAMNLFIGGSVHAAGAYTSPYPQNAR-NSVVYLDTAVQEGYTWFGSASMKPIGRVEVELFDDTVPITARNFRELCRGYQNKTP**

**TcCyP25**  **--------MVYLTRLVRRVHRGPIRNIFSYPLNEVNP---------VVFFEITVEG----------DALGQVTIELFHDTVPKTSENFRSLCTGERG--F**

110 120 130 140 150 160 170 180 190 200

....|....|....|....|....|....|....|....|....|....|....|....|....|....|....|....|....|....|....|....|

**CyPD_human** **-G--FGYKGSTFHRVIPSFMCQAGDFTNHNGTGGKSIYGSRFPDENFT---LKHVGPGVLSMANAGPNTNGSQFFICTIKTDWLDGKHVVFGHVKEGMDV**

**TcCyP21**  **-G--FGYKGSIFHRVIRNFMIQGGDFTNFDGTGGKSIYGTRFDDENLK---IKHF-VGAVSMANAGPNSNGSQFFVTTAPTPWLDGRHVVFGKVVEGMDV**

**TcCyP22**  **SGKALCYKGSKFHRVIPQFMCQGGDFTNGNGTGGESIYGMKFPDESFAGRAGKHFGPGTLSMANAGPNTNGSQFFICTAATEWLDGKHVVFGQITKGYEV**

**TcCyP24**  **EGKPLHYKGSVFHRIIPGFMIQGGDITKGNGTGGCSIYGVRFKDESFDSKAGAHKGPGILSMANAGRNTNGSQFFICTVSCPWLDGKHVVFGQVLRGFDH**

**TcCyP25**  **IQCPLYYKGIPFHRIIPGFIVQGGDILTKDGRGNVSVFGFPFLDESFKGKAGKHL-PGTVAMAHSGPNQNGSQFFFNMRRNEHLDGKYVVCGQVLEGWDL**

210 220 230 240 250 260

....|....|....|....|....|....|....|....|....|....|....|....|. **% identity % positives**

**CyPD_human** **VKKIESFGS-KSGRTSKKIVITDCGQLS---------------------------------**

**TcCyP21**  **VKKVENTKTGLNDKPKKAVKINDCGVL----------------------------------** **54 66**

**TcCyP22**  **IEKVEANGS-RSGATRQPILITDCGEVKNN-------------------------------** **65 77**

**TcCyP24**  **VKAIEEVGT-PHGKPSKTVLVSDCGVLKEAS------------------------------** **56 63**

**TcCyP25**  **VERVAALCGSRCGVPVSRAWITDCGQSSGAKLEEAQRALCGERALHSMPGKEVLDLISPRY** **48 65**
