# Supplementary material for: A homolog of cyclophilin D is expressed in Trypanosoma cruzi and is involved in the oxidative stress–damage response
Source: Cell Death Discov. 2017 Feb 6;3:16092–. doi: 10.1038/cddiscovery.2016.92 (PMC5292771; doi:10.1038/cddiscovery.2016.92)
Supplement: Supplementary Table S1 [file cddiscovery201692-s2.doc]

**Supplemental Table S1**

|  | **iPSORT** | | **MitoProt II** | |
| --- | --- | --- | --- | --- |
|  | Signal peptide | Mitochondrial Localization Signal | Cleavage site | Probability of M.E. |
| *Tc*CYP19 | No | No | NP | 0.0708 |
| *Tc*CYP20 | No | No | Yes | 0.4068 |
| ***Tc*CYP21** | **Yes** | **No** | **Yes** | **0.4702** |
| ***Tc*CYP22** | **No** | **Yes** | **Yes** | **0.8334** |
| ***Tc*CYP24** | **Yes** | **No** | **Yes** | **0.4109** |
| ***Tc*CYP25** | **No** | **Yes** | **NP** | **0.9979** |
| *Tc*CYP28 | No | No | NP | 0.1886 |
| *Tc*CYP30 | No | No | NP | 0.0586 |
| *Tc*CYP35 | No | No | NP | 0.3660 |
| *Tc*CYP35.3 | No | No | Yes | 0.5791 |
| *Tc*CYP40 | No | No | NP | 0.0901 |
| *Tc*CYP110 | No | No | NP | 0.0956 |
